# Self-reported frequency of handwashing among pet and non-pet owners in different situations: results of four surveys of the general adult population in Germany

**DOI:** 10.1186/s12889-024-21106-3

**Published:** 2024-12-24

**Authors:** Karolin M. E. Nettelrodt, Thomas von Lengerke

**Affiliations:** Hannover Medical School (MHH), Centre of Public Health, Department of Medical Psychology, Carl-Neuberg-Str. 1, Hannover, 30625 Germany

**Keywords:** Hand hygiene, Overestimation bias, Behavioral indicators, Compliance, Infection prevention/control, Population surveillance

## Abstract

**Background:**

Zoonotic diseases are partly associated with pets. However, data is sparse on pet owners’ compliance with preventive recommendations. Also, research focuses on self-reports, which are subject to overestimation biases, i.e., assessing one’s actual performance to be better than it is. One reason is task difficulty: people tend to overestimate their performance on hard tasks. Regarding handwashing, compliance after touching animals should be harder for pet vs. non-pet owners due to the number of opportunities. This study tests for differences in self-reported handwashing between pet and non-pet owners, and explores reasons for non-compliance. Thus, it aims to provide insights on how to improve self-report behavioral assessment methods in public health and One Health research.

**Methods:**

Data from cross-sectional computer-assisted telephone surveys of the general population in Germany aged 16–85 years in 2012, 2014, 2017 and 2019 were analyzed (*N* = 15,559; response rate: 45.9%). Handwashing frequency was operationalized for nine indications using the item “How often do you wash your hands in each of the following situations: ‘never/almost never‘, ‘seldom‘, ‘mostly‘, ‘always/almost always‘?”, with the latter defining compliance. In 2017 and 2019, those reporting to ‘never/almost never‘ or ‘rarely‘ wash hands were questioned regarding possible reasons. Chi²-tests, Cohen’s d’s and multiple logistic regressions were used.

**Results:**

Pet and non-pet owners differed in self-reported handwashing compliance primarily in the indication “After touching animals” (35.5% vs. 55.7%, effect size: d = 0.45). For other indications (e.g., “After using the toilet”), differences were insignificant (≤|3.6%|, d ≤ 0.11). Additionally, 79% of pet owners who rarely or almost never washed their hands after touching animals felt it is not necessary (non-pet owners: 67.1%; d = 0.34). Reporting to not have an appropriate washing facility available was rarer among pet owners (44.5% vs. 63%, d = 0.41). Differences regarding other reasons were trivial (d ≤ 0.16), including “It takes too long” (16.9 vs. 13.3%; *p* = .138 in multiple regression).

**Conclusions:**

Study limitations include that due to unknown true compliance, over- and underestimations have to be inferred. Yet, that the only substantial difference between pet and non-pet owners pertained to „After touching animals” suggests such effects. While pet owners obviously adjust for task difficulty, the likely residual overestimation should be reduced by measures using script-based covert recall or survey items with response categories constructed to better resemble subjective compliance ratios.

**Supplementary Information:**

The online version contains supplementary material available at 10.1186/s12889-024-21106-3.

## Highlights

Self-reports of handwashing compliance in the population tend to be biased.

One such bias is overestimation, especially regarding tasks with high difficulty.

Pet (vs. non-pet) owners report lower handwashing compliance after touching animals.

No differences affect other situations, e.g. after using the toilet or handshaking.

Assessment scales should integrate definitions of compliance to increase validity.

**Supplementary Information:**

The online version contains supplementary material available at 10.1186/s12889-024-21106-3.

## Introduction

Zoonoses are infectious diseases caused by pathogens that are naturally transmissible between vertebrate animals and humans and, with over 200 known types, represent an increasingly important and widespread threat to global health [1, 2]. For instance, in Europe the first and second most reported zoonoses in 2022 were campylobacteriosis and salmonellosis, respectively [3], and in Germany, the prevalence of Lyme disease, which is caused by borrelia bacteria, after tick bites has been estimated to be 20% [4]. At the same time, compared to other animal hosts, pets have received comparatively little attention regarding the zoonotic risks associated with their ownership and husbandry, and the prevention of these risks [5]. Yet, based on existing evidence it is fair to assert that a substantial proportion of zoonotic transmissions and diseases in humans is associated with pets [5–24].

At the same time, data is sparse on pet owners’ compliance with recommendations on zoonoses prevention, which can be classified into pet health and husbandry, types and ages of pets, and personal hygiene [25]. For instance, according to the German Federal Centre for Health Education (Bundeszentrale für gesundheitliche Aufklärung, BZgA), pet owners are recommended to wash their hands after any physical contact with their pet or it’s feeding bowl [26]. However, in 2019 only 45% of German pet owners self-reportedly washed their hands more than 10 times a day, which only slightly exceeded the rate among respondents not owning a pet (41%) [27]. Westgarth et al. found a self-reported compliance rate of 96% among dog owners in terms of always or usually washing their hands after picking up dog feces (58% after touching dogs) [28]. At the same time, there are virtually no data on hand hygiene behavior in the general population based on direct observation: the only estimates of rates of handwashing with soap based on such methods come from Freemann et al. and Wolf et al. and range from 19 to 26% for the indication “after potential fecal contact” [29–30]. Neither study included data specific to pet owners or data from Germany (though Wolf et al. estimated an overall rate of 48.7% for this country using a modeling approach [30]). A similar assertion pertains to a more recent systematic review of interventions to change hand hygiene and mask use behavior during the COVID-19 pandemic [31]. Thus, research on the prevention of pet-associated infections still has to rely on self-reports of personal hygiene behaviors both in the general population and in at-risk groups such as persons under 5 or over 64 years of age, immunosuppressed individuals, and pregnant women [10, 25].

This reliance on self-reports presumes an at least acceptable level of validity, if the goal is to assess actual behavior. However, a meta-analysis found that 79% of the variance in the association between self-reported and objectively assessed pro-environmental behaviors remained unexplained [32]. One major bias, especially in questionnaires using rating scales, is that people often over-report their own behavior. This phenomenon is generally termed overestimation and represents one facet of overconfidence [33]. A systematic review of the concordance between observed and self-reported protective behavior among members of the public during COVID-19 found that self-reports overestimated observed compliance by up to a factor of five [34]. At the same time, due to their acceptability and practicability, self-reports still are likely to remain an important method of behavioral assessment. Thus, it is essential to gain theoretical and empirical insight into the sources of overconfidence in general and of overestimation in particular.

In this context, motivational and cognitive factors can be distinguished [35]. Among the former, the most commonly cited drivers of inflated self-assessments are desires to view and present oneself positively or better than others [35]. Among cognitive factors, a key determinant that contributes to overestimation when judging one’s own performance in a given task is argued to be its difficulty [36]. Specifically, people “… tend to overestimate their performance on hard tasks and underestimate it on easy tasks” [33, p. 3]. The mechanism behind this pattern is that when one has imperfect knowledge of one’s true performance level (which usually is the case), the subjective estimation of one’s performance will regress to the overall mean, i.e., the mean across all levels of difficulty. This implies that for a task with a relatively low average achievement rate, i.e., a difficult task, a given subjective estimate will exceed this rate, while underestimation will occur in the case of easy tasks [37].

Considering the task “washing one’s hands after physical contact with a pet”, high levels of compliance (defined as the proportion of opportunities in which hands are washed divided by all opportunities in which handwashing is indicated) should be much more difficult to achieve for pet owners than non-pet owners. This is due to the high frequency of these contacts, and justifies the hypothesis that pet owners may overestimate their compliance, while non-pet owners will tend to underestimate it. As task difficulty translates to questionnaire item difficulty [38], pet and non-pet owners should be likely to differ in their agreement to a response category which indicates high compliance of handwashing after physical contact with a pet. Most probably, pet owners should be more likely to agree that they wash their hands consistently. At the same time, handwashing compliance for other indications, e.g., after using the toilet, may not be prone to such variance between these two groups because of the lack of such systematic differences in the frequency of situation-specific handwashing opportunities.

Against this background, we used data of the BZgA’s four general population-representative surveys on hygiene and infection control conducted between 2012 and 2019 [27, 39–41] to test for differences in self-reported handwashing between pet and non-pet owners across different situations in which handwashing is recommended [26, 42]. Additionally, we explore reasons for not washing one’s hands after touching animals which were assessed in the latter two surveys (2017 and 2019) [27, 41]. By doing so, the study aims to provide insights informed by the psychology of survey response on how to improve self-report behavioral assessment methods in public health and One Health research.

## Materials and methods

### Study design, setting, and participants

The four surveys on hygiene and infection control commissioned by the BZgA and conducted in 2012, 2014, 2017 and 2019 were administered by the Forsa Institute for Social Research and Statistical Analysis as cross-sectional, representative, computer-assisted telephone interview surveys of the general population in Germany aged 16–85 years 2019 [27, 39–41]. They used a dual-frame multi-stage random sampling design based on selection frameworks for fixed-line numbers and mobile phone numbers by the Working Group of German Market and Social Research Institutes [43]. Within households reached via fixed-line, the person included was selected by the last birthday method. i.e., interviewing the eligible person within the household who had the most recent birthday. The samples were increased to include sufficient sub-samples of pregnant women in 2012 and 2014 and of parents with children under the age of 16 in the household in 2017 and 2019, respectively. In total, *N* = 4,483 interviews were conducted in 2012, *N* = 4,491 in 2014, *N* = 4,018 in 2017, and *N* = 4,001 in 2019. Data sets for 2012 and 2014 were publicly available online at the Data Archive for the Social Sciences of the GESIS Leibniz Institute for the Social Sciences [44, 45], while those for 2017 and 2019 were provided by the BZgA upon our request (Andrea Rückle, personal communication, August 23, 2023). All four data sets were pooled using IBM SPSS Statistics v28, resulting in a total of *N* = 16,993 interviews. *N* = 1,311 interviews were excluded from statistical analyses: five respondents who stated not washing their hands even once a day, 146 respondents who did not provide any information about handwashing frequency or duration, 1,058 respondents who did not provide information on handwashing frequency in the nine indications to be examined in our analysis (see below, Sect. 2.2), 88 respondents without information on their age and two respondents without information on their gender, and twelve respondents due to missing information on pet ownership. Eventually, 15,682 respondents were included. In statistical analyses, data was weighted to compensate for sampling biases inherent in the differential selection probabilities for the two sampling frames (fixed-line and mobile) and the oversampling of pregnant women and parents with children under 16. Weighting resulted in a sample with rounded total of *N* = 15,559.

### Measures: survey items and compliance indices

Only items consistent in wording and answer categories across all four surveys were used for analyses, with one exception for frequency of handwashing in indications: in 2012 and 2014 answer categories were “almost never”, “seldom”, “mostly” and “almost always”, whereas in 2017 and 2019 the end points were labelled “never or almost never” and “always or almost always”. In the following sections, our translations of the original German survey items are provided. The original items are available from the corresponding author and in [27, 39–41]. For all items, the answer categories “I don’t know” and “Not specified” were not presented in the interview but coded either if the respondent gave a respective answer by him- or herself, or responded in a way that after could, after clarification, be fitted validly into one of these categories by the interviewer.

#### Handwashing compliance

Frequency of handwashing was operationalized for nine indications using the following item: “How often do you wash your hands in each of the following situations, i.e., “never/almost never”, “seldom”, “mostly”, or “always/almost always”?” Indications were “Before eating”, “After touching animals”, “After handshaking”, “Before handling food”, “After coming home from outside”, “After using the toilet”, “After blowing one’s nose or coughing in one’s hand”, “After being with someone who had the flu, a gastrointestinal disease, or a similarly contagious disease”, and “Before visiting someone who is weakened by illness”. Following [46], handwashing compliance was defined for each variable as “almost always or always”.

#### Socio-demographics

Indexing algorithms followed socio-demographic standards by the German Federal Statistical Office [47] and are available upon reasonable request from the corresponding author and in [46]. Gender, owning a pet and having a chronic disease were assessed by single items. The item for pet ownership read as follows: “Do you have pets?”, and the item regarding chronic disease “Do you suffer from a chronic disease?” (as examples for diseases the survey participants may personally suffer from, chronic respiratory diseases, heart or circulatory diseases, liver and kidney diseases, neurological diseases, metabolic diseases such as diabetes, and congenital or acquired immune deficiencies were given). Age was calculated as difference between the survey date and the birthday reported by the respondent and was grouped into categories (16–29, 30–44, 45–59 and 60–85 years). Educational background was coded based on the highest achieved or pursued school or university degree. Migration background was coded with “yes” if the country of birth of both the respondent and his parents was not Germany and/or their citizenship was not German. Having children under 16 years of age was coded based on reported size of the household and for non-single households coding if such children lived there. For having children aged five or younger the survey date and reported child’s children’s year(s) of birth were taken into account. Those survey respondents stating they were employed, in vocational training, higher education or on parental/maternal leave were asked if they personally worked in the medical field with patient contact. Answering “yes” was coded as “currently working in healthcare”.

#### Reported reasons for non-compliance

In the 2017 and 2019 surveys, participants reporting to “never or almost never” or “rarely” wash their hands given an indication were presented a list of five possible reasons for their behavior and were asked if each reason applied to them or not. Multiple selections were possible. The proposed reasons were “I feel that it is not necessary”, “I do not have an appropriate washing facility available”, “I do not think of it, or forget it”, “It takes too long” and “Others might consider it inappropriate”.

### Statistical analysis

Chi-Square-tests with odds ratios (OR) and 95%-confidence intervals (95%-CI) were used to test for differences between pet and non-pet owners. Breslow-Day-tests were used to test for heterogeneity of the ORs across surveys. *P*-values < 0.05 were considered to signify statistical significance. Additionally, ORs were transformed into Cohen’s d-coefficients [48] in order to assess effect sizes of the differences. Multiple logistic regression analyses were conducted to adjust for socio-demographic and other potential confounders of the associations of pet ownership with self-reported handwashing and with the reasons for (almost) never or rarely washing one’s hands. These variables were those being associated with pet ownership, i.e., gender, age, educational background, migration background, having children < 16 in household, having a chronic disease, and currently working in healthcare (see Table 1).

## Results

### Sample description

Table 1 shows that overall there were *N* = 5,738 pet owners (36.9%), whose distribution across the four surveys was not significantly unequal (*p* = .144). Compared to non-pet owners, they were significantly more likely to be female (55.2% vs. 48.3%) and 59 years of age or younger (80% vs. 67.3%). They less often had higher educational background (29.2% vs. 34.4%), and more often intermediate educational background (34.2% vs. 29.3%). They were less likely to report any migration background (15.9% vs. 22.5%), and more likely to have children under 16 years in their household (31.4% vs. 18.9%). The latter did not pertain to children under 6 years of age (9.2% vs. 9.3%). Finally, significant but percentage-wise smaller differences pertained to reporting to have at least one chronic disease (30.6% vs. 28%) and currently working in the healthcare sector with contact to patients (vs. not: 10.9% vs. 8.7%).

**Table 1:** Sample characteristics, overall and stratified for non-pet owners and pet owners

			Total *N* = 15,559				Non-pet owners (*N* = 9,821, 63.1%**)				Pet owners (5,738, 36.9%**)				Chi²-test***		
			N		%*		N		%*		N		%*		Chi²		*p*
*Year of survey*															5.4		= 0.144
	2012		4,100		26.4%		2,585		26.3%		1,515		26.4%				
	2014		4,111		26.9%		2,566		26.1%		1,545		26.9%				
	2017		3,704		23.8%		2,313		23.6%		1,391		24.2%				
	2019		3,644		23.4%		2,357		24.0%		1,287		22.4%				
*Gender*															70.5		< 0.001
	Men		7,651		49.2%		5,082		51.7%		2,569		44.8%				
	Women		7,908		50.8%		4,739		48.3%		3,169		55.2%				
*Age (in years)*															298.6		< 0.001
	16–29		3,140		20.2%		1,913		19.5%		1,227		21.4%				
	30–44		3,652		23.5%		2,142		21.8%		1,510		26.3%				
	45–59		4,409		28.3%		2,554		26.0%		1,855		32.3%				
	60–85		4,358		28.0%		3,212		32.7%		1,146		20.0%				
*Educational background* ^a^															56.7		< 0.001
	Higher		5,016		32.5%		3,347		34.4%		1,669		29.2%				
	Medium		4,808		31.1%		2,856		29.3%		1,952		34.2%				
	Lower		5,624		36.4%		3,530		36.3%		2,094		36.6%				
*Migration background*															97.5		< 0.001
	No		12,430		79.9%		7,607		77.5%		4,823		84.1%				
	Yes		3,126		20.1%		2,211		22.5%		915		15.9%				
*Children under 16 in household*															312.7		< 0.001
	No		11,808		76.5%		7,898		81.1%		3,910		68.6%				
	Yes		3,628		23.5%		1,839		18.9%		1,789		31.4%				
*Children age 5 or younger in household*															0.04		0.845
	No		13,924		90.7%		8,786		90.7%		5,138		90.8%				
	Yes		1,422		9.3%		901		9.3%		521		9.2%				
*Chronic disease* ^*b*^															12.01		< 0.001
	No		11,029		71.1%		7,052		72.0%		3,977		69.4%				
	Yes		4,490		28.9%		2,738		28.0%		1,752		30.6%				
*Currently working in healthcare* ^*c*^															20.2		< 0.001
	No		14,068		90.5%		8,961		91.3%		5,107		89.1%				
	Yes		1,485		9.5%		858		8.7%		627		10.9%				

*Notes.* * Column percentages ** Row percentages *** Pet ownership by row variable ^a^ Higher educational background equals upper secondary school, middle educational background equals intermediate school, and lower educational background equals secondary general school.

^b^ Chronic respiratory diseases, heart or circulatory diseases, liver and kidney diseases, neurological diseases, metabolic diseases such as diabetes, and congenital or acquired immune deficiencies were given as examples for diseases the survey participants may personally suffer from ^c^ Pertains to the survey participants personally

### Pet owners’ and non-pet owners’ self-reported handwashing behavior in different situations

**Fig. 1:**
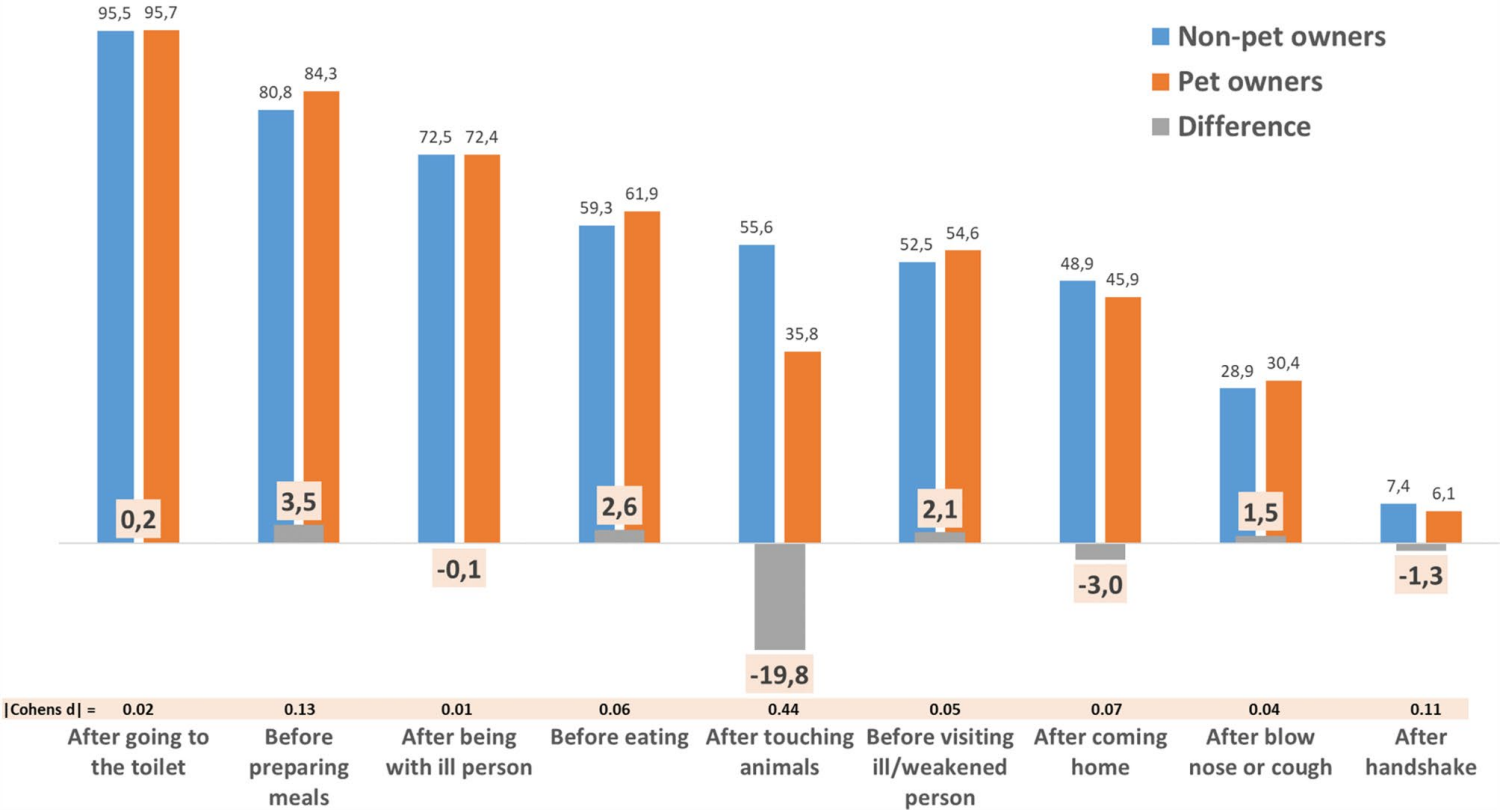
Self-reported handwashing compliance of pet owners and non-pet owners as proportions of those reporting to always or almost always wash their hands in nine different indications (in %)

Figure 1 shows the compliance rates of pet and non-pet owners for the nine different situations in which handwashing is indicated. The indications are arranged in descending order from left to right by the overall compliance rates, which can be found in the appendix (Table A.1), correspond to the non-pet owners’ pattern, and range from “after using the toilet” with 95.7% to “after handshaking” with 6.9%. As Fig. 1 visualizes, with one exception differences between pet and non-pet owners were in lower single digits, with values ranging from 3.6% for “Before handling food” to -0.3% for “After being with someone with an infectious disease”. The exception is the indication “After touching animals”: here, the compliance rate for pet owners was 20.2% lower than that of non-pet owners. Detailed statistics including Chi²-tests and Breslow-Day tests (testing for heterogeneity in differences between pet and non-pet owners across surveys) can be found in the appendix (Table A.1). The Breslow-Day test for “After touching animals” shows that the differences between pet and non-pet owner did not differ across surveys (*p* = .237). By conventional classifications of effect sizes in terms of Cohen’s d, which were calculated based on the odds ratios, all differences were trivial (d < 0.20) except for “After touching animals”, in regard to which a small effect tending towards medium range was found (d = 0.45). As the multiple logistic regression analyses shown in Table A.2 in the appendix reveal, this effect is independent of gender, age, educational background, migration background, children under 16 in the household, chronic disease, and working in healthcare. Adjusting for these factors, the odds ratio for pet ownership is 0.43 (95%-CI: 0.40; 0.46, *p* < .001), corresponding to an effect size of d = 0.47. The mean number of indications for which compliance was reported was 4.9 among pet owners and 5 among non-pet owners (not shown). While being statistically significant (F(1,14954) = 17.4, *p* < .001), this difference was trivial in terms of its effect size (d = 0.08)

### Pet and non-pet owners’ reasons for rarely or (almost) never washing their hands after touching animals

**Fig. 2:**
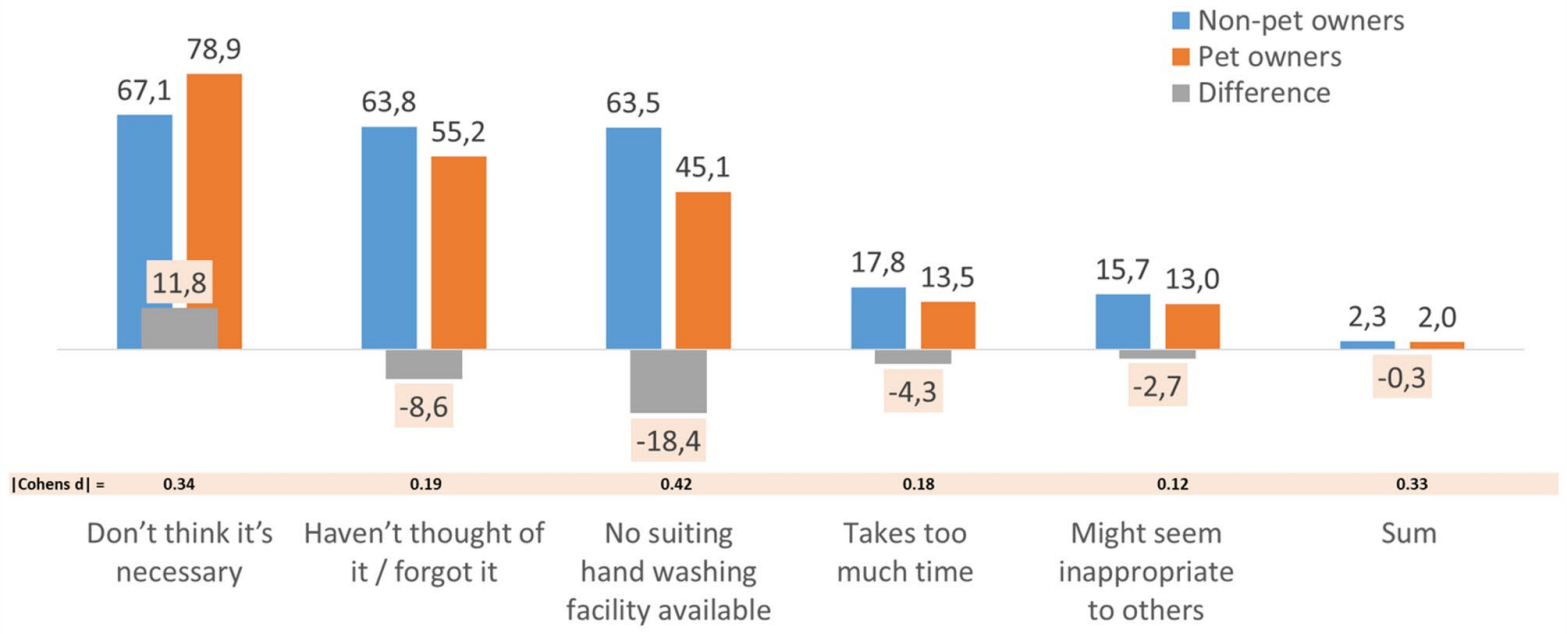
Reasons for self-reportedly never, almost never or rarely washing one’s hands among pet owners and non-pet owners (as proportions – in % – of those confirming each reason)

Those *N* = 1932 respondents in the 2017 and 2019 surveys who reported to “never or almost never” or “rarely” wash their hands after touching animals were asked regarding reasons for this. The strongest agreement rate pertained to “I feel it is not necessary” (73.5%), followed by “I do not think of it, or forget it” (58.6%), “I do not have an appropriate washing facility available” (53%), “It takes too long” (15%), and “Others might consider it inappropriate” (13.6%; see appendix, Table A.3). As Fig. 2 shows, pet owners were 11.9% more likely than non-pet owners to feel that it is not necessary to wash their hands in such situations. In contrast, they were less likely to check the other reasons, most prominently lack of an appropriate washing facility (-18.5%), followed by not thinking of or forgetting to wash hands (-7.3%), that it takes too long (-3.6%), and that others might consider it inappropriate (-3%). Detailed statistics can be found in Table A.3 in the appendix. In terms of effect sizes, all differences were trivial (d < 0.20) except that for “I feel it is not necessary” and “Others might consider it inappropriate”, representing small effects (d = 0.34 and d = 0.41, respectively). As multiple logistic regression analyses show, these two effects were independent of gender, age, educational background, migration background, children under 16 years of age in the household, chronic disease, and working in healthcare (see appendix, Table A.4). Adjusting for these factors, the odds ratios were 1.93 (95%-CI: 1.54–2.41, *p* < .001) for “I feel it is not necessary” and 0.46 (95%-CI: 0.38–0.56, *p* < .001) for “Others might consider it inappropriate”, which correspond to effect sizes of d = 0.36 and 0.43. Regarding the reason “It takes too long”, the difference between pet and non-pet owners became statistically insignificant (OR = 0.82, 95%-CI 0.62; 1.07, *p* = .138). The mean number of reasons given was 2 among pet owners and 2.1 among non-pet owners (not shown), a difference that was insignificant (F_(1,1786)_ = 3.3, *p* = .072) and trivial (d = 0.09).

## Discussion

Results can be summarized as follows. First, pet owners and non-pet owners differed in terms of their self-reported handwashing compliance primarily in regard to the indication “After touching animals”. Specifically, pet owners’ compliance was 35.5%, and thus 20.2% lower than that of non-pet owners (effect size: d = 0.45). For all other eight indications, differences were trivial (≤|3.6%|, d ≤ 0.11). In multiple regression analysis, this pattern was found to be independent of potential confounders. Second, 79% of pet owners who had indicated to rarely or almost never wash their hands after touching animals reported to do so because they feel it is not necessary, while among non-pet owners, these were 67.1% (d = 0.34). In contrast, the proportion of respondents reporting that they do not have an appropriate washing facility available was lower among pet owners (44.5%) than non-pet owners (63%, d = 0.41). Differences regarding the other three reasons presented in the surveys were trivial (d ≤ 0.16). Among others, for indicating that it takes too long (16.9% among pet vs. 13.3% among non-pet owners), pet ownership as a predictor emerged to be insignificant in the multiple regression analysis (*p* = .138).

Before further discussion, strengths and limitations of the present study have to be considered. Commissioned by a national governmental specialist authority (BZgA), data are representative of the German adult population. Also, with a five-digit sample size the study is reasonably large, and variations across four years spanning almost one decade could be accounted for. Finally, while previous studies focused on pet owners’ compliance to hygiene recommendations generally (e.g [28, 50–54]). , we compared pet and non-pet owners’ handwashing specifically after touching animals.

Regarding limitations, first of all handwashing behavior was self-reported, which implies that there is no way of telling the actual frequency of handwashing by the respondents, also due to the tendencies to misestimate one’s own behavior outlined in the introduction. This would presuppose data including both observed and self-reported behavior, whereby self-reports could be validated and calibrated (much like self-reported body weight and height have been on the basis of data from examinations) [55]. Thus, all conclusions drawn from the present data have to consider that while self-reports may correlate with actual behavior, degrees of over- and under-estimation remain speculative.

Second, we focused on handwashing frequency and neglected both the two other facets of handwashing, i.e., duration and technique [42, 46], and hand disinfection with alcohol-based handrub. While this partly was due to these facets and behaviors not being comprehensively assessed in all surveys (technique was only assessed in 2012), the main reason was that only frequency was assessed for different indications including “After touching animals”, making contrasts between different situations possible for this facet only.

Third, while we – following [46] – defined compliance as “always or almost always” washing one’s hands, and thus contrasted it with “often”, “seldom”, and “never or almost never”, reasons for non-compliance in the 2017 and 2019 surveys were assessed for those respondents only who had reported to “seldom” or “never or almost never” wash their hands in given situations. Thus, results on reasons for non-compliance are only valid for these two levels of lower frequency of handwashing as self-reported by respondents.

Fourth, the items for pet ownership and handwashing included no definition on what constituted a “pet”, which type of pet(s) were owned by the households, whether the pet(s) lived inside or outside the home, and whether handwashing occurred after pet-specific tasks such as handling pet treats and food, picking up waste, or cleaning litterboxes and equipment such as cages and aquariums. Also, the method of selecting respondents within households, i.e., the last birthday method, implies that it remains unknown whether individual respondents were those household members with the most pet contact/responsibilities or not.

Fifth, regional differences, e.g., urban vs. suburban vs. rural contexts could not been addressed in the present analysis. However, in an earlier publication no significant differences between West and East Germans were found in handwashing behavior other than before eating (54 vs. 66%, respectively [27]). Regarding cultural differences, the present results of mostly higher self-reported compliance among respondents with a migration background, which includes the indication “after touching animals” (see Table A.2 in the appendix), are consistent with the pattern found in 2023 [56].

Finally, the present analysis used data on handwashing from 2012 to 2019 primarily in order to scrutinize basic issues concerning self-reports as an assessment instrument for behavior. Thus, the COVID-19 pandemic and its potential impact on handwashing behaviors (be it self-reported or observed) is obviously not reflected in the present data and conclusions drawn from it. However, given data showing notable stability of handwashing compliance from April 2020 to February 2021 in two countries comparable to Germany (Belgium and France) [57], this bias may be negligible – especially as other measures such as wearing masks were more focal. Also, in 2023 the proportions of pet and non-pet-owner Germany who (almost) always wash their hands (29% vs. 55% [56]) has practically not changed compared to 2019 (31.5% vs. 54.4%; see appendix, Table A.1).

With these limitations in mind, results can be rationalized as follows. The biggest difference by far between pet and non-pet owners was found for the indication “After touching animals”, with pet owners having 2.3 times *lower* odds to report washing their hands almost always or always. Note that this does *not* automatically imply that pet owners *under*estimate their performance. The reason is that, as noted, their true level of compliant behavior is unknown. Consider the following scenario: Assuming pet owners on average touch an animal 1,500 times per month (i.e., 50 times per day), the self-reported compliance rate of 35.5% “always almost” or “always” would represent a total of 3,055,485 compliant handwashing procedures in our sample, i.e., an average of 533 per pet owner. Further assume that the *real* numbers are 860,700 compliant opportunities in total, i.e., 150 per pet owner, and thus a compliant rate of 10%. This would imply an overestimation of 25.5%. In contrast, assume that among non-pet owners there are only nine animal contacts per month, which would – based on the 55.7% who stated to (almost) always wash their hands in this group – imply 49,233 compliant opportunities, i.e. five per person. Finally, in case the real compliance rate is 66.7%, i.e., 58,926 compliant opportunities in total and six per person, this would imply an underestimation of 11%. Of course, this is just a speculative arithmetic example. Since to the best of our knowledge there is no empirical data on the actual numbers of handwashing opportunities (be they compliant or non-compliant) among pet vs. non-pet owners, no definite conclusions can be drawn regarding the degree of over- and/or underestimation (if any).

Additionally, other explanations have to be considered. On the one hand, “After touching *animals*” may be interpreted differently by pet vs. non-pet owners. Pet owners may exclusively think of their pet(s), thus underrepresenting farm and/or wild animals, while non-pet owners may “exclusively” think of the latter two. Also, “After *touching* animals” may be understood by some simply in terms of stroking the fur of pets such as dogs, cats, or petting zoo animals, while others may have professional contact with animals, e.g. in agriculture, and think of feeding or cleaning them as well. On the other hand, pet owners may see their pets as family members and tend to humanize them [58]. This type of anthropomorphism, which may imply lesser need to comply with handwashing recommendations due to low perceived risks of pathogen transmission, also may include sharing one’s bed with pets or allowing them to lick one’s face to demonstrate positive emotions, and has been linked to increased zoonotic risks [59]. The fact that pet owners in the present study more frequently than non-pet owners confirmed “I feel it is not necessary” as a reason for seldomly or (almost) never washing their hands after touching animals may point in this direction.

At the same time, the empirical pattern that the single substantial difference between pet vs non-pet owners pertained to the indication „After touching animals” remains. Combined with the probably valid assumption that handwashing opportunities for the other indications are (much more) evenly distributed across pet vs. non-pet owner both in terms of frequency and the difficulty of complying, it seems obvious to assume that misestimation effects are involved here. The difference between the two groups not tending in the hypothesized direction may point to the possibility that pet owners do partly adjust for task difficulty. This suggests that scales such as those used here, i.e., using different and specified indications, generate reduced overestimation due to this very specificity.

As mentioned, respondents reporting to seldomly or (almost) never washing their hands after touching animals were presented with five possible reasons that they could confirm or disconfirm. The reason most frequently confirmed by both pet and non-pet owners was “I feel that it is not necessary”, with a higher agreement among pet owners. For all other reasons, agreement was more frequent for non-pet owners. In particular, this pertained to “I do not have an appropriate washing facility available”, possibly due to pet owners higher levels of preparedness for situations such as handling their pets outdoors. “It takes too long” and “Others might consider it inappropriate” were more rarely confirmed by both groups. Especially regarding the former, this is intriguing because this reason at least implicitly is one rationale for the basic argument that high handwashing compliance after touching animals should be much more difficult to achieve for pet than for non-pet owners, i.e., due to the frequency of this situation and thus the high expenditure of time needed for high compliance. Together with the higher disagreement both to the necessity of handwashing and the unavailability of facilities among pet owners, this suggests they perceive relatively low risks associated with non-compliance.

Regarding public health implications, for interventions targeting pet owners in order to prevent zoonotic transmission the present study suggests to sensitize them to the possibility of overestimating one’s handwashing behavior, and thus the risks positively biased illusions compromising compliance [60]. Also, evidence-based risk communication [61] is warranted to increase the perceived need of hygienic behavior to prevent infections such as zoonoses. Finally, specific suggestions for improving the validity of future research that relies on self-reports are given in the following concluding section.

## Conclusion

Self-reports of behaviors such as handwashing are susceptible to biases due to motivational and cognitive factors. As long as there is insufficient knowledge and understanding of these biases, e.g., overestimation, the evaluation of self-reported compliance rates will always remain uncertain as to whether and, if at all, how exact actual compliance is measured. This can lead to conclusions that do not reflect reality, especially when comparing compliance rates, e.g., between different groups or different indications. Direct observation remains the gold standard or at least a key approach to assess hand hygiene compliance [62, 63]. While it is costly and resource intensive, it provides the least biased results (though not unbiased: [64, 65]), and has been implemented in professional settings such as hospitals. For people in their everyday domestic settings and situations, such as pet owners’ interactions with their pets, it is largely considered unfeasible and potentially even unethical. Thus, alternative approaches to measuring hand hygiene compliance such as script-based covert recall methods [66, 67] and the day reconstruction method [68] have been described, which essentially represent self-reports, but are designed to assist respondents to provide more realistic assessments of their behavior [69]. In contrast, the present study used more traditional scaling, showing that there was some adjustment for task difficulty, but overestimation is still likely. One implication for assessing handwashing behavior by self-reports in questionnaires may be to use two items: one estimating the number of opportunities in which handwashing is indicated per unit of time, and one estimating the number of these opportunities in which handwashing is actually performed. Alternatively and at the very least, the rate of handwashing when indicated should be assessed directly, i.e., in percent like in [60, 70, 71]. Thus, respondents would either ex- or implicit be prompted to take into account not only the numerators of “their” compliance ratios, but also the denominators. This may improve the validity of self-reports as vagueness due to variation in the understanding of terms such as “almost always” or “seldom” would be reduced, and response categories would better resemble subjective compliance ratios.

Finally, practical implications pertain to health professionals both in human and veterinary medicine. Given the knowledge-behavior-gap in personal hygiene behavior [72] and effective interventions going beyond motivation by including self-regulatory mechanisms both for laypeople [73–75] and professionals [76–78], integrated motivational and self-regulatory interventions such as trainings action planning skills should be developed to promote pet husbandry-related hygiene compliance. Also, many information materials are currently delivered primarily online, thus relying on high-distance, population-level modes of delivery. While these may be effective especially if adhering to evidence-base standards of risk communication [61] and behavior change [79], they should be supplemented by face-to-face-mediated formats, e.g. motivational interviewing and brief action planning, to be administered by credible sources such as pet owners’ primary care physicians or veterinarians [54].

## Electronic Supplementary Material

Below is the link to the electronic supplementary material.

Supplementary Material 1

## Data Availability

The data in this study were gathered in telephone surveys commissioned by the German Federal Centre for Health Education (Bundeszentrale für gesundheitliche Aufklärung, BZgA), a specialist authority within the portfolio of the German Federal Ministry of Health. The surveys were administered by the Forsa Institute for Social Research and Statistical Analysis [23, 35-37] on behalf of the BZgA. Data sets from 2012 to 2014 are publicly available online at the Data Archive for the Social Sciences of the GESIS Leibniz Institute for the Social Sciences [40, 41], and those for 2017 and 2019 were provided by the BZgA upon our request (Dr. Andrea Rückle, personal communication, August 23, 2023).
