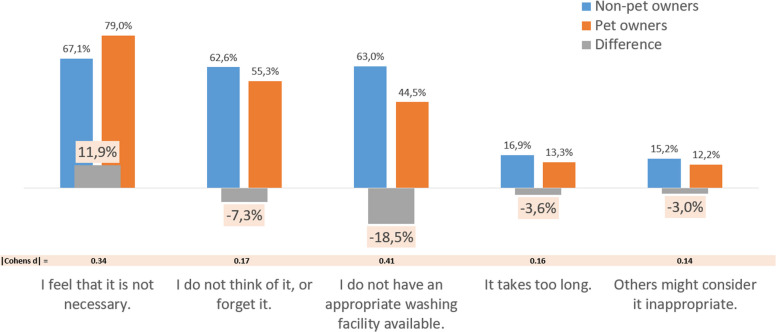# Correction: Self-reported frequency of handwashing among pet and non-pet owners in different situations: results of four surveys of the general adult population in Germany

**DOI:** 10.1186/s12889-025-25949-2

**Published:** 2026-02-05

**Authors:** Karolin M. E. Nettelrodt, Thomas von Lengerke

**Affiliations:** Hannover Medical School (MHH), Centre of Public Health, Department of Medical Psychology, Carl-Neuberg-Str. 1, Hannover, 30625 Germany

**Correction** **:** **BMC Public Health 24, 3581 (2024)**

**https://doi.org/10.1186/s12889-024–21106-3**

Following publication of the original article [1], the authors identified errors in Figures 1 and 2. The correct figures are given below.

Incorrect Figure 1


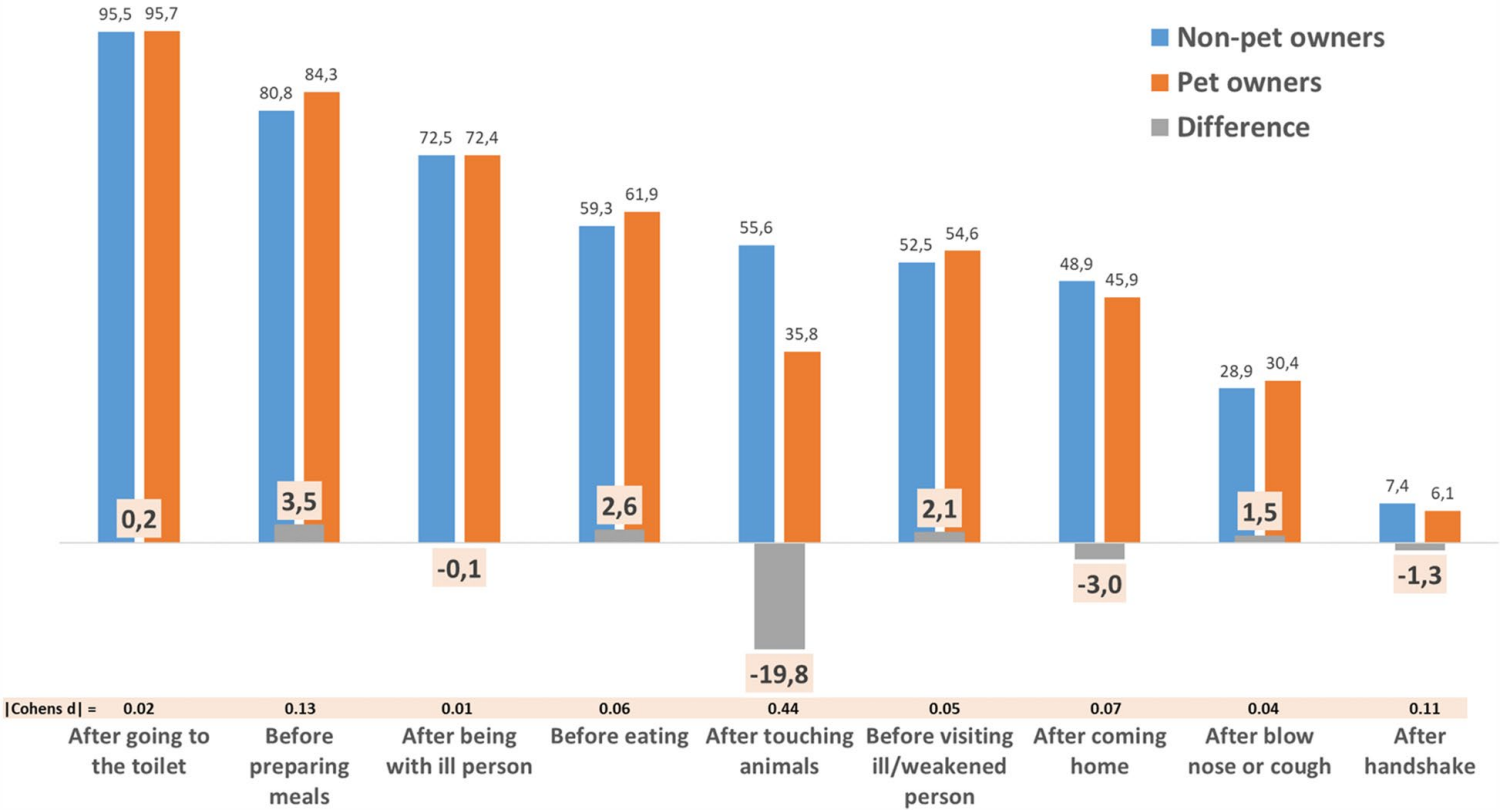


Correct Figure 1


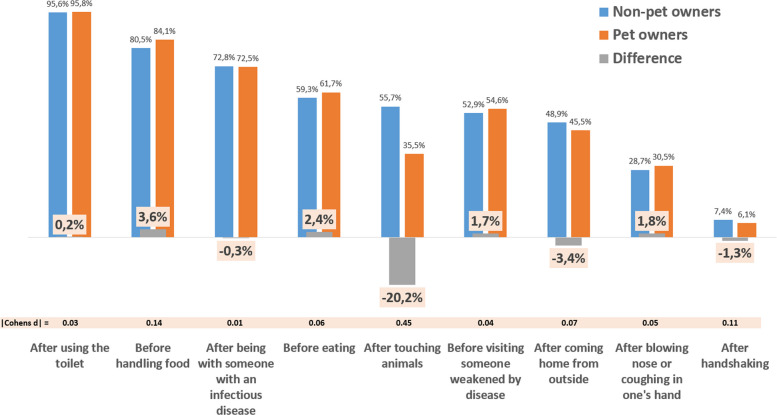


Incorrect Figure 2


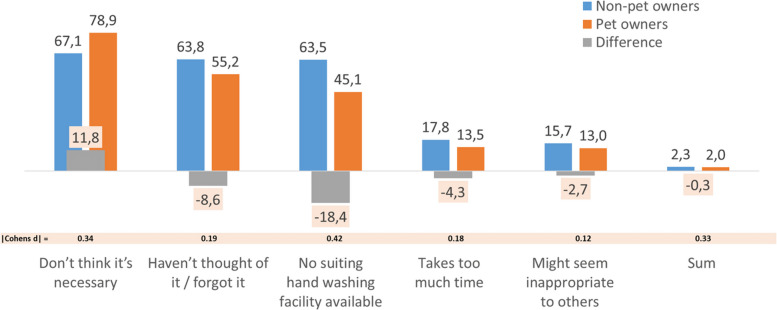


Correct Figure 2