# Supplementary material for: Self-reported frequency of handwashing among pet and non-pet owners in different situations: results of four surveys of the general adult population in Germany
Source: BMC Public Health. 2024 Dec 24;24:3581. doi: 10.1186/s12889-024-21106-3 (PMC11669229; doi:10.1186/s12889-024-21106-3)
Supplement: Supplementary file 1 — Supplementary Material 1 [file 12889_2024_21106_MOESM1_ESM.pdf]

Table A.1 Self-reported handwashing compliance as proportions (in %) of those reporting to always or almost always wash their hands, for nine different indications (overall and for pet owners and non-pet owners) [§]

	Total		Non-pet owners		Pet owners		Δ[a]	Chi²-test				Breslow-Day-Test[a]
	N	%	N	%	N	%		Chi²	*p*	OR	95% CI	Chi²
**After using the toilet**												
**Overall**	**14,886**	**95.7%**	**9,388**	**95.6%**	**5,498**	**95.8%**	**0.2%**	**0.35**	**0.554**	**1.05**	**0.89; 1.23**	
2012	3,963	96.7%	2,498	96.6%	1,465	96.7%	0.1%	0.13	0.911	1.02	0.72; 1.45	
2014	3,876	94.3%	2,402	93.6%	1,474	95.4%	1.8%	5.56	0.018	1.41	1.06; 1.88	7.8,
2017	3,537	95.5%	2,209	95.5%	1,328	95.4%	-0.1%	0.02	0.886	0.98	0.71; 1.34	p=0.050
2019	3,510	96.3%	2,279	96.7%	1,231	95.6%	-1.1%	2.55	0.110	0.75	0.53; 1.07	
**Before handling food**												
**Overall**	**12,734**	**81.8%**	**7,910**	**80.5%**	**4,824**	**84.1%**	**3.6%**	**30.5**	**<0.001**	**1.28**	**1.17; 1.39**	
2012	3,450	84.1%	2,140	82.8%	1,310	86.5%	3.7%	9.71	0.002	1.33	1.11; 1.59	
2014	3,306	80.4%	2,011	78.4%	1,295	83.8%	5.4%	18.18	<0.001	1.43	1.21; 1.69	4.8,
2017	3,015	81.4%	1,870	80.8%	1,145	82.3%	1.5%	1.24	0.266	1.10	0.93; 1.31	p=0.185
2019	2,963	81.3%	1,889	80.1%	1,074	83.4%	3.3%	6.11	0.013	1.25	1.05; 1.50	
**After being with someone with an infectious disease**												
**Overall**	**11,311**	**72.7%**	**7,153**	**72.8%**	**4,158**	**72.5%**	**-0.3%**	**0.22**	**0.637**	**0.98**	**0.91; 1.06**	
2012	2,851	69.6%	1,796	69.5%	1,055	69.7%	0.2%	0.02	0.890	1.01	0.88; 1.16	
2014	2,904	70.6%	1,818	70.8%	1,086	70.3%	-0.5%	0.15	0.703	0.97	0.85; 1.12	0.2,
2017	2,795	75.4%	1,751	75.7%	1,044	75.1%	-0.6%	0.18	0.673	0.97	0.83; 1.13	p=0.977
2019	2,761	75.8%	1,788	75.9%	973	75.6%	-0.3%	0.03	0.863	0.99	0.84; 1.12	

Table A.1 cont.

	Total		Non-pet owners		Pet owners		Δ[a]	Chi^2-test				Breslow-Day-Test[a]
	N	%	N	%	N	*Chi^2*		Chi^2	*p*	OR	95% CI	Chi^2
**Before eating**												
**Overall**	9,364	60.2%	**5,824**	**59.3%**	**3,540**	**61.7%**	**2.4%**	**8.70**	**0.003**	**1.11**	**1.03; 1.18**	
2012	2,644	64.5%	1,595	61.7%	1,049	69.3%	7.6%	23.99	<0.001	1.40	1.22; 1.60	
2014	2,437	59.3%	1,534	59.8%	903	58.4%	-1.4%	0.71	0.399	0.95	0.83; 1.08	21.6,
2017	2,260	61.0%	1,380	59.7%	880	63.3%	3.6%	4.74	0.030	1.16	1.02; 1.34	p<0.001
2019	2,023	55.5%	1,315	55.8%	708	55.0%	-0.8%	0.21	0.651	0.97	0.85; 1.11	
**After touching animals**												
**Overall**	**7,506**	**48.3%**	**5,467**	**55.7%**	**2,039**	**35.5%**	**-20.2%**	**588.3**	**<0.001**	**0.44**	**0.41; 0.47**	
2012	2,067	50.4%	1,482	57.3%	585	38.6%	-18.7%	133.9	<0.001	0.47	0.41; 0.53	
2014	1,960	47.7%	1,414	55.1%	546	35.3%	-19.8%	151.9	<0.001	0.45	0.39; 0.51	4.2,
2017	1,790	48.3%	1,288	55.7%	502	36.1%	-18.6%	133.99	<0.001	0.45	0.39; 0.52	p=0.237
2019	1,689	46.4%	1,283	54.4%	406	31.5%	-22.9%	175.4	<0.001	0.39	0.33; 0.45	
**Before visiting someone weakened by illness**												
**Overall**	**8,324**	**53.5%**	**5,193**	**52.9%**	**3,131**	**54.6%**	**1.7%**	**4.20**	**0.041**	**1.07**	**1.01; 1.14**	
2012	2,015	49.1%	1,277	49.4%	738	48.7%	-0.7%	0.18	0.671	0.97	0.86; 1.10	
2014	2,037	49.5%	1,221	47.6%	816	52.8%	5.2%	10.56	0.001	1.23	1.09; 1.40	7.1,
2017	2,146	57.9%	1,329	57.5%	817	58.7%	1.2%	0.58	0.446	1.05	0.92; 1.21	p=0.068
2019	2,126	58.3%	1,366	57.9%	760	59.1%	1.2%	0.43	0.512	1.05	0.91; 1.20	

Table A.1 cont.

	Total		Non-pet owners		Pet owners		Δ[a]	Chi²-test				Breslow-Day-Test[a]
	N	%	N	%	N	%		Chi²	*p*	OR	95% CI	Chi²
**After coming home from outside**												
**Overall**	**7,412**	**47,6%**	**4,801**	**48.9%**	**2,611**	**45.5%**	**-3.4%**	**16.70**	**<0.001**	**0.87**	**0.82; 0.3**	
2012	2,006	48.9%	1,288	49.8%	718	47.4%	-2.4%	2.26	0.132	0.91	0.80; 1.03	
2014	1,877	45.7%	1,201	46.8%	676	43.8%	-3.0%	3.66	0.056	0.88	0.78; 1.00	0.9,
2017	1,826	49.3%	1,172	50.7%	654	47.0%	-3.7%	4.64	0.031	0.86	0.76; 0.99	p=0.818
2019	1,703	46.7%	1,140	48.4%	563	43.7%	-4.7%	7.14	0.008	0.83	0.72; 0.95	
**After blowing nose or coughing in one's hand**												
**Overall**	**4,563**	**29.3%**	**2,816**	**28.7%**	**1,747**	**30.5%**	**1.8%**	**5.50**	**0.019**	**1.09**	**1.01; 1.17**	
2012	1,241	30.3%	759	29.4%	482	31.8%	2.4%	2.72	0.099	1.12	0.98; 1.29	
2014	1,104	26.9%	656	25.6%	448	29.0%	3.4%	5.78	0.016	1.19	1.03; 1.37	6.5,
2017	1,127	30.4%	717	31.0%	410	29.5%	-1.5%	0.95	0.329	0.93	0.81; 1.08	p=0.091
2019	1,091	29.9%	684	29.0%	407	31.6%	2.6%	2.69	0.101	1.13	0.98; 1.31	
**After handshaking**												
**Overall**	**1,077**	**6.9%**	**728**	**7.4%**	**349**	**6.1%**	**-1.3%**	**9.90**	**0.002**	**0.81**	**0.71; 0.92**	
2012	313	7.6%	209	8.1%	104	6.9%	-1.2%	2.00	0.157	0.84	0.66; 1.07	
2014	210	5.1%	146	5.7%	64	4.1%	-1.6%	4.76	0.029	0.72	0.53; 0.97	5.7,
2017	311	8.4%	218	9.4%	93	6.7%	-2.7%	8.47	0.004	0.69	0.54; 0.89	p=0.129
2019	243	6.7%	155	6.6%	88	6.8%	-0.2%	0.09	0.762	1.04	0.80; 1.37	

Notes: [§] Differences to results in [49], which were based on the 2012 and 2014 surveys, due to slightly different inclusion criteria [a] Percentage difference between pet and non-pet owners

Table A.2 Results of multiple logistic regression analyses for handwashing compliance in different situations (indications)§

	After using the toilet			Before handling food			After being with someone with an infectious disease			Before eating			After touching animals			Before visiting someone weakened by illness			After coming home from outside			After blowing nose or coughing in one's hand			After handshaking		
	OR	95%-CI	p	OR	95%-CI	p	OR	95%-CI	p	OR	95%-CI	p	OR	95%-CI	p	OR	95%-CI	p	OR	95%-CI	p	OR	95%-CI	p	OR	95%-CI	p
**Pet ownership**																											
Yes	0.98	0.83-1.15	= 0.774	1.16	1.06-1.27	= 0.001	0.95	0.88-1.03	= 0.221	1.12	1.04-1.20	= 0.002	0.43	0.40-0.46	< 0.001	1.00	0.94-1.08	= 0.916	0.88	0.82-0.95	< 0.001	1.02	0.95-1.10	= 0.568	0.83	0.72-0.95	= 0.007
No	ref.			ref.			ref.			ref.			ref.			ref.			ref.			ref.			ref.		
**Gender**																											
Women	1.90	1.61-2.25	< 0.001	1.65	1.51-1.80	< 0.001	1.70	1.58-1.83	< 0.001	1.22	1.14-1.31	< 0.001	1.46	1.37-1.57	< 0.001	1.53	1.43-1.63	< 0.001	1.77	1.66-1.90	< 0.001	1.73	1.60-1.86	< 0.001	1.21	1.06-1.37	= 0.005
Men	ref.			ref.			ref.			ref.			ref.			ref.			ref.			ref.					
**Age**																											
60-85 years of age	0.72	0.56-0.92	= 0.008	0.82	0.72-0.94	= 0.004	1.33	1.19-1.49	< 0.001	0.99	0.89-1.10	= 0.840	1.44	1.30-1.61	< 0.001	1.19	1.08-1.32	< 0.001	1.20	1.08-1.33	< 0.001	0.78	0.69-0.87	< 0.001	0.80	0.64-1.00	= 0.046
45-59 years of age	1.01	0.79-1.30	= 0.909	0.87	0.77-0.99	= 0.038	1.34	1.21-1.49	< 0.001	1.24	1.12-1.36	< 0.001	1.85	1.67-2.04	< 0.001	1.26	1.14-1.38	< 0.001	1.22	1.11-1.35	< 0.001	0.78	0.70-0.86	< 0.001	1.09	0.89-1.32	= 0.414
30-44 years of age	0.83	0.65-1.07	= 0.143	0.70	0.62-0.80	< 0.001	1.66	1.49-1.86	< 0.001	1.35	1.22-1.50	< 0.001	2.06	1.85-2.29	< 0.001	1.25	1.13-1.38	< 0.001	1.51	1.36-1.67	< 0.001	0.71	0.64-0.80	< 0.001	1.34	1.10-1.64	= 0.004
16-29 years of age	ref.			ref.			ref.			ref.			ref.			ref.			ref.			ref.			ref.		
**Educational background****																											
lower	0.58	0.48-0.71	< 0.001	1.31	1.18-1.45	< 0.001	1.05	0.96-1.15	= 0.334	1.52	1.40-1.65	< 0.001	1.31	1.21-1.43	< 0.001	1.25	1.16-1.36	< 0.001	1.17	1.08-1.27	< 0.001	1.88	1.71-2.06	< 0.001	1.82	1.54-2.14	< 0.001
intermediate	1.10	0.88-1.37	= 0.426	1.31	1.18-1.46	< 0.001	1.17	1.07-1.28	< 0.001	1.25	1.56-1.36	< 0.001	1.25	1.15-1.36	< 0.001	1.25	1.15-1.36	< 0.001	1.05	0.97-1.15	= 0.209	1.37	1.25-1.51	< 0.001	1.17	0.98-1.40	= 0.089
higher	ref.			ref.			ref.			ref.			ref.			ref.			ref.			ref.			ref.		
**Migration background**																											
Yes	0.68	0.58-0.81	< 0.001	1.07	0.96-1.19	= 0.239	0.92	0.84-1.01	= 0.085	1.60	1.46-1.74	< 0.001	1.28	1.18-1.40	< 0.001	0.99	0.91-1.07	= 0.771	1.41	1.30-1.54	< 0.001	1.43	1.31-1.56	< 0.001	1.43	1.23-1.66	< 0.001
No	ref.			ref.			ref.			ref.			ref.			ref.			ref.			ref.			ref.		
**Children under 16 years of age in household**																											
Yes	0.95	0.77-1.17	= 0.617	0.87	0.78-0.98	= 0.021	0.92	0.84-1.01	= 0.089	0.85	0.78-0.93	< 0.001	0.94	0.86-1.03	= 0.167	0.95	0.88-1.04	= 0.283	1.00	0.92-1.09	= 0.970	0.85	0.77-0.93	< 0.001	1.08	0.90-1.30	= 0.412
No	ref.			ref.			ref.			ref.			ref.			ref.			ref.			ref.			ref.		
**Chronic disease**																											
Yes	1.30	1.01-1.56	= 0.006	1.11	1.01-1.22	= 0.036	1.15	1.06-1.25	= 0.001	1.09	1.02-1.18	= 0.019	1.17	1.09-1.26	< 0.001	1.07	0.99-1.15	= 0.090	1.06	0.99-1.15	= 0.098	1.02	0.94-1.11	= 0.611	1.03	0.90-1.19	= 0.636
No	ref.			ref.			ref.			ref.			ref.			ref.			ref.			ref.			ref.		
**Currently working in healthcare**																											
Yes	0.90	0.67-1.20	= 0.453	0.93	0.80-1.08	= 0.336	1.56	1.36-1.80	< 0.001	1.15	1.02-1.29	= 0.021	0.93	0.83-1.04	= 0.190	1.63	1.45-1.83	< 0.001	0.98	0.88-1.10	= 0.763	1.50	1.33-1.68	< 0.001	1.29	1.04-1.59	= 0.022
No	ref.			ref.			ref.			ref.			ref.			ref.			ref.			ref.			ref.		

Note: §OR: odds ratio; CI: confidence interval; all estimates are from the multiple logistic regression model for each respective behavioral indicator, which included all predictors listed **lower: secondary general school, intermediate: middle school, higher: upper secondary school

Table A.3 Participants' reasons for (almost) never or rarely washing hands after touching animals*,**

	Total		Non-pet owners		Pet owners		Δ[a]	Chi²-test				Breslow-Day-Test[a]
	N	%	N	%	N	%		Chi²	*p*	OR	95% CI	Chi²
**I feel that it is not necessary.**												
Overall	1,413	73.5%	597	67.1%	816	79.0%	11.9%	34.83	<0.001	1.85	1.50; 2.27	
2017	666	73.5%	259	65.1%	407	80.1%	15.0%	25.93	<0.001	2.16	1.60; 2.92	2.01, p=0.156
2019	747	73.5%	338	68.7%	409	77.9%	9.2%	11.04	<0.001	1.61	1.21; 2.13	
**I do not think of it, or forget it.**												
Overall	1,130	58.7%	558	62.6%	572	55.3%	-7.3%	10.70	0.001	0.74	0.61; 0.89	
2017	520	57.3%	239	59.9%	281	55.3%	-4.6%	1.92	0.166	0.83	0.64; 1.08	1.3, p=0.251
2019	610	59.9%	319	64.8%	291	55.2%	-9.6%	9.80	0.002	0.67	0.52; 0.86	
**I do not have an appropriate washing facility available.**												
Overall	1,021	53.0%	561	63.0%	460	44.5%	-18.5%	65.59	<0.001	0.47	0.39; 0.57	
2017	516	56.8%	263	65.9%	253	49.7%	-16.2%	23.95	<0.001	0.51	0.39; 0.67	0.98, p=0.320
2019	505	49.7%	298	60.6%	207	39.4%	-21.2%	45.41	<0.001	0.42	0.33; 0.55	
**It takes too long.**												
Overall	288	15.0%	151	16.9%	137	13.3%	-3.6%	5.10	0.024	0.75	0.58; 0.96	
2017	148	16.3%	67	16.8%	81	15.9%	-0.9%	0.12	0.732	0.94	0.66; 1.34	3.5, p=0.062
2019	140	13.8%	84	17.1%	56	10.7%	-6.4%	8.78	0.003	0.58	0.40; 0.83	
**Others might consider it inappropriate.**												
Overall	262	13.6%	136	15.2%	126	12.2%	-3.0%	3.82	0.051	0.77	0.59; 1.00	
2017	131	14.4%	71	17.8%	60	11.8%	-6.0%	6.52	0.011	0.62	0.43; 0.90	2.6, p=0.109
2019	131	12.9%	65	13.2%	66	12.6%	-0.6%	0.09	0.770	0.95	0.66; 1.37	

Notes. *Multiple responses were possible. ** Reasons only available for participants of survey 2017 and 2019 who stated to never, almost never or seldomly wash hands after touching an animal;
[a] Percentage difference between pet owners and non-pet owner

Table A.4 Results of multiple logistic regression analyses for reasons for (almost) never or rarely washing ones hands after touching animals§

	I feel that it is not necessary.			I do not think of it, or forget it.			I do not have an appropriate washing facility available.			It takes too long.			Others might consider it inappropriate.		
	OR	95%-CI	p	OR	95%-CI	p	OR	95%-CI	p	OR	95%-CI	p	OR	95%-CI	p
**Pet ownership**															
Yes	1.93	1.54-2.41	< 0.001	0.78	0.64-0.95	= 0.013	0.46	0.38-0.56	< 0.001	0.82	0.62-1.07	= 0.138	0.74	0.56-0.98	= 0.035
No	ref.			ref.			ref.			ref.			ref.		
**Gender**															
Women	1.14	0.91-1.43	= 0.270	1.19	0.98-1.45	= 0.088	0.85	0.70-1.04	= 0.113	0.89	0.67-1.17	= 0.398	1.31	0.98-1.75	= 0.066
Men	ref.			ref.			ref.			ref.			ref.		
**Age**															
60-85 years of age	1.09	0.78-1.53	= 0.604	0.61	0.46-0.81	< 0.001	0.83	0.63-1.09	= 0.174	0.45	0.31-0.64	< 0.001	1.22	0.84-1.77	= 0.291
45-59 years of age	0.57	0.42-0.78	< 0.001	0.40	0.31-0.53	< 0.001	1.02	0.78-1.34	= 0.880	0.33	0.22-0.48	< 0.001	0.63	0.43-0.93	= 0.019
30-44 years of age	0.76	0.55-1.05	= 0.096	0.50	0.37-0.68	< 0.001	0.76	0.57-1.01	= 0.060	0.43	0.29-0.64	< 0.001	0.42	0.27-0.65	< 0.001
16-29 years of age	ref.			ref.			ref.			ref.			ref.		
**Educational background****															
lower	0.73	0.56-0.96	= 0.023	1.16	0.91-1.49	= 0.230	0.94	0.74-1.20	= 0.631	1.21	0.87-1.69	= 0.253	2.80	1.96-4.00	< 0.001
intermediate	1.07	0.82-1.40	= 0.622	0.94	0.75-1.18	= 574	1.11	0.88-1.39	= 0.370	0.91	0.66-1.25	= 0.545	1.66	1.17-2.35	= 0.004
higher	ref.			ref.			ref.			ref.			ref.		
**Migration background**															
Yes	0.54	0.42-0.70	< 0.001	1.61	1.25-2.07	< 0.001	0.98	0.77-1.24	= 0.835	0.56	0.39-0.81	= 0.002	1.00	0.71-1.40	= 0.979
No	ref.			ref.			ref.			ref.			ref.		
**Children under 16 years of age in household**															
Yes	0.88	0.65-1.19	= 0.418	1.08	0.84-1.39	= 0.545	1.09	0.85-1.39	= 0.511	0.79	0.57-1.11	= 0.173	1.90	1.29-2.80	= 0.001
No	ref.			ref.			ref.			ref.			ref.		
**Chronic disease**															
Yes	0.68	0.54-0.86	= 0.001	0.86	0.70-1.07	= 0.171	1.58	1.27-1.96	< 0.001	0.66	0.48-0.92	= 0.014	0.60	0.42-0.84	= 0.003
No	ref.			ref.			ref.			ref.			ref.		
**Currently working in healthcare**															
Yes	1.12	0.74-1.69	= 0.595	0.66	0.47-0.93	= 0.016	1.00	0.71-1.40	= 0.990	0.57	0.32-1.01	= 0.056	0.52	0.29-0.96	= 0.035
No	ref.			ref.			ref.			ref.			ref.		

Note: § OR: odds ratio; CI: confidence interval; all estimates are from the multiple logistic regression model for each respective behavioral indicator, which included all predictors listed **lower: secondary general school, intermediate: middle school, higher: upper secondary school
